# The Treatment of Melioidosis 

**DOI:** 10.3390/ph3051296

**Published:** 2010-04-27

**Authors:** Timothy J.J. Inglis

**Affiliations:** 1 Division of Microbiology & Infectious Diseases, PathWest Laboratory Medicine WA, QEII Medical Centre, Nedlands, WA 6009, Australia; E-Mail: tim.inglis@health.wa.gov.au; Tel.: +61-8-9346-3461; Fax: +61-8-9381-7139; 2 Microbiology & Immunology, School of Biomedical, Biomolecular and Chemical Sciences, Faculty of Life & Applied Sciences, University of Western Australia, Crawley, WA 6009, Australia; 3 School of Pathology and Laboratory Medicine, Faculty of Medicine & Dentistry, University of Western Australia, Crawley, WA 6009, Australia

**Keywords:** melioidosis, treatment, antibiotics, adjunct therapy

## Abstract

Melioidosis is a complex bacterial infection, treatment of which combines the urgency of treating rapidly fatal Gram negative septicaemia with the need for eradication of long-term persistent disease in pulmonary, soft tissue, skeletal and other organ systems. Incremental improvements in treatment have been made as a result of multicentre collaboration across the main endemic region of Southeast Asia and northern Australia. There is an emerging consensus on the three main patterns of antimicrobial chemotherapy; initial (Phase 1) treatment, subsequent eradication (Phase 2) therapy and most recently post-exposure (Phase 0) prophylaxis. The combination of agents used, duration of therapy and need for adjunct modalities depends on the type, severity and antimicrobial susceptibility of infection. New antibiotic and adjunct therapies are at an investigational stage but on currently available data are unlikely to make a significant impact on this potentially fatal infection.

## 1. Introduction

Melioidosis is a potentially fatal infection caused by the Gram negative bacillus, *Burkholderia pseudomallei*, following an encounter with contaminated soil or surface water. Like other environmental bacteria, *B. pseudomallei* is highly resistant to many antibiotics. The antibiotics normally used for first line treatment of Gram negative septicaemia (e.g., ampi-/amoxycillin, aminoglycosides, ceftriaxone) are ineffective in the treatment of melioidosis, highlighting the need for recognition of melioidosis when presumptive antimicrobial therapy is commenced. In other words, the decision to treat melioidosis and the choice of antimicrobial agent may have to be taken before any laboratory results are available to guide clinical decision-making. Lengthy courses of treatment are required, including a prolonged eradication phase.

**Table 1:** Melioidosis treatment summary.

Application	Agent	Amount *	Route	Frequency	Duration	Variations	References
**Phase 0**: post-exposure prophylaxis							
Within 24 hr of high- probability exposure ^a^	trimethoprim-sulphamethoxazole	320:1600 mg	p.o.	12 hourly	3 weeks ^a^	amoxicillin/ clavulanic acid if allergic to trimethoprim-sulpha-methoxazole	[30,31]
**Phase 1**: acute & severe infection, induction stage							
Alternative agents for primary therapy	Ceftazidime	2g	i.v. ^b^	8 hourly	≥ 14 days	4-8 weeks for deep infection	[1,2,3,5]
	OR Meropenem	1g (2g for C.N.S. infection	i.v.	8 hourly	≥ 14 days		[2,6]
	OR Imipenem	1g	i.v.	8 hourly	≥ 14 days		[5]
Adjunct therapy for deep-seated focal infection	AND trimethoprim-sulphamethoxazole	320:1600 mg	p.o. ^c^	12 hourly	≥ 14 days	for neurological, prostatic, bone, joint infections	[1,2,3]
	AND folic acid	5 mg	p.o.	daily			
	AND considerG-CSF ^d^	263 μg	s.c.	daily	3 days	Within 72 hrs of admission	[20,21]
Step-down combination for outpatient or extension clinic use	Ceftazidime	6 g in 240 mL Normal saline	i.v.	24 hour infusion	2-4 weeks	For hospital in the home (HITH)	[9]
	AND trimethoprim-sulphamethoxazole	320:1600 mg	p.o.	12 hourly			
**Phase 2**: eradication stage							
2 of , in order of preference, after Phase 1 or for primary use in superficial infections	trimethoprim-sulphamethoxazole	320;1600 mg	p.o.	12 hourly	≥ 3 months^e^	Subject to antibiotic susceptibility	[13]
	doxycycline	100 mg	p.o.	12 hourly	≥ 3 months		[13]
	amoxicillin/ clavulanic acid	500/125 mg	p.o.	8 hourly	≥ 3 months		[14,15]
	folic acid	5 mg	p.o.	daily	≥ 3 months	With trimethoprim-sulphamethoxazole	[30]

* doses may require adjustment in renal failure ^a^ suggested by expert consensus, but lacks trial-based clinical evidence; ^ b^ doses provided as guide only based on 70kg male; ^c^ i.v. = intravenous, p.o. = oral, ^d^ G-CSF = granulocyte- colony stimulating factor; ^e^ some recommend 5 months eradication therapy.

## 2. Therapeutic Guidelines

There have been several attempts to formulate clinical guidelines for clinicians faced with patients who might have melioidosis [1,2]. One in particular reviewed the then available literature for evidence of efficacy, and addressed the more difficult dilemmas in clinical therapeutics including duration of treatment, the value of combination therapy, the need for prolonged eradication therapy and the role of other treatment modalities [1]. The key recommendations were use of the cephalosporin Ceftazidime or a carbapenem antibiotic for initial treatment of acute infection over 2-4 weeks and a combination of co-trimoxazole and doxycycline for eradication over a 12-20 week period. Broadly similar recommendations set in the context of diagnostic and clinical review of Australian experience were adapted for use in the emerging disease setting of South and Central America [2]. More recently, those recommendations were updated by an Australian group noted for clinical trials on melioidosis therapy [3]. The key features of these guidelines and other recommendations reviewed here are summarized in Table 1.

## 3. Antibiotic Choice

The antibiotics used to treat melioidosis fall into two distinct categories: (a) those suitable for the treatment of the acute septicaemia phase of disease (phase 1), and (b) those used in the subsequent eradication phase of therapy, previously known as "maintenance" therapy (phase 2). 

### 3.1. Phase 1, acute infection

The principal choice of agents used in the first phase comprises a cephalosporin, usually Ceftazidime, or a carbapenem, usually Meropenem. Alternatives such as cefepime and imipenem have both been proposed for acute therapy, and Imipenem has been successfully used in clinical practice [4,5]. There has been a recent shift from Ceftazidime to Meropenem in some centres [6] due to concerns over the risk of Ceftazidime resistance and poor cellular bioavailability, compared with the good intracellular penetration of carbapenem antibiotics. Though reduced intracellular efficacy was demonstrated in an *in vitro* model [7], laboratory studies do not yet indicate a high level of antibiotic resistance in *B. pseudomallei* to any of the agents used to treat the acute phase (phase 1) of infection [8]. Ceftazidime can be administered as a continuous infusion via an elastomeric device, and has produced satisfactory outcomes when used to continue Phase 1 therapy of acute melioidosis as outpatient therapy in remote Australian communities [9]. Concerns about early relapse of septicaemic infection have led some to recommend combination of co-trimoxazole with Ceftazidime to augment the latter’s bactericidal effect. This has been shown to be unnecessary [10], and there is a case report of the combination having a potentially antagonistic effect on intracellular *B. pseudomallei* [11]. The same report highlights a possible additive effect of simultaneous use of a Carbapenem and a fluoroquinolone agent, such as Ciprofloxacin, subject to initial antimicrobial susceptibility testing. Where decisions on use of co-trimoxazole are likely to be informed by antimicrobial susceptibility testing, susceptibility should be assessed in the laboratory by an MIC method such as e-test, rather than disk diffusion or break point. No prospective randomised trial of Ceftazidime *versus* meropenem has been conducted yet, so the question of which antimicrobial agent has superior efficacy in acute infection remains open. What is a little more clear is the duration of treatment, which needs to be a minimum of two weeks in patients with a positive blood culture [1,7], though such a short course would be exceptional and restricted to patients without any of the recognised invasive disease-promoting co-morbidities of diabetes, chronic renal failure and alcoholic liver disease or any indicators of deep-seated infection, secondary dissemination or predictably slow response to therapy. More commonly, patients receive 3-4 weeks of intravenous therapy and close monitoring of their clinical progress. At the other extreme, patients with disease severe enough to require intensive care (e.g. multiple organ systems failure) may need longer intravenous antibiotic therapy, and can be cured of life-threatening septicaemia with multiple organ failure by aggressive and co-coordinated management [11]. 

### 3.2. Phase 2, eradication therapy

Conversion to eradication antibiotic therapy needs careful supervision because this is the point at which some patients are at risk of septicaemic relapse [12]. It is common practice to briefly overlap initial intravenous and eradication antibiotic therapy in order to assess tolerance of the oral eradication agents, though this dual approach is unnecessary if co-trimoxazole has been used throughout Phase 1. There has been considerable discussion of the best combination of agents. Co-trimoxazole and doxycycline is a suitable combination therapy for eradication [13], and co-trimoxazole alone has been used successfully for eradication therapy in the Northern Territory of Australia. Co-amoxyclav has been widely used in combination with one or more of the others but is unsuitable for single use and is may be counterproductive, particularly when given at suboptimal dose intervals [14]. Consensus guidelines for use of amoxicillin/clavulanic acid as a second line agent have been developed recently [15]. Previous use of the term 'maintenance therapy' to describe this phase of melioidosis chemotherapy was misleading. The aim is better rendered by describing it as “eradication”, since the purpose is to completely remove any residual infection that might relapse at a later date. The mechanism of relapse after prolonged sequestration or dormancy of *B. pseudomallei* in tissue macrophages and other privileged sites is poorly understood. A proportion of these infections may in fact be due to re-infection, rather than relapse [16]. Nevertheless, application of an aggressive two-phase approach to treatment reduces the risk of relapse occurring, as was shown recently in Malaysia where a state melioidosis registry had the unexpected effect of reducing the relapse rate [17]. A period of 12-20 weeks eradication therapy is now widely used with good evidence for efficacy [18], but a small number of patients may require a longer period of Phase 2 eradication treatment. These are patients with extensive underlying co-morbidity in which complete eradication is an unrealistic goal, particularly when the disease is multifocal and unresponsive to antimicrobial chemotherapy. Some of these patients may in fact have re-infections rather than true relapses. Given the potential implications for patient management of these two possible explanations for a second *B. pseudomallei* infection, a scoring system has been proposed to help distinguish melioidosis relapse from re-infection [19].

## 4. Other Therapeutic Agents

The baseline melioidosis mortality rate of 35% reported in Thailand and 19% in Australia has led some centres to consider the use of complementary, non-antibiotic interventions in severe septicaemic infections [1,3,11]. Granulocyte colony stimulating factor (G-CSF) has been tried as an early intervention in severe acute melioidosis in an uncontrolled retrospective study performed in Northern Australia [20]. Disappointing results were demonstrated in a recent randomised controlled trial of G-CSF for the treatment of septicaemic melioidosis in Thailand, although longer survivals were observed in individual cases [21]. Other potential adjunctive agents for which there is less clinical efficacy data include human recombinant activated protein C [22], which has been used in other forms of Gram negative septicaemia, in order to modify the host response to overwhelming infection. There is growing interest in a potential role for statins in the management of acute melioidosis, following the incidental observation that patients with systemic bacterial infection fare better if they receive statin treatment [23]. *In vitro* studies into statin activity on *B. pseudomallei* are at an early stage. There is as yet no published clinical experience specific to melioidosis. A recent critical review of clinical trials of statin use in sepsis found a modest and inconsistent effect on clinical outcome [24], and highlights how difficult it will be to assess the impact of this family of non-antibiotic agents on the early stages of a sporadic infection. It is likely that statins will find a place in the treatment of melioidosis only after a more extensive theoretic and *in vitro* case has been built for their use and assessed in the setting of randomized clinical trials.

## 5. Unresolved Issues

Treatment of melioidosis is a challenge even where there are adequate resources to support patients with multiple organ failure and extensive clinical experience. In resource-poor settings, the cost of optimal Phase 1 and 2 therapy imposes severe restraints and is a likely contributor to unsuccessful clinical outcomes. The duration of the phase 1 of antimicrobial therapy is longer than for most other infections. Pressure on hospital bed occupancy has led to evaluation of a hospital-in-the-home approach which has been successfully used in northern Australia [9]. However, the longer phase 2 of treatment has its own specific challenges, such as compliance and monitoring. Clinical progress assessment and laboratory tests suitable for the first phase of treatment are not as helpful by this stage. C-reactive protein has been used in this role for many years [25], but is unreliable as a sole indicator of imminent relapse [26]. If the earlier phase of initial therapy has been successful, disease will be subclinical; its persistence entirely at a tissue and cellular level. This partly explains the preference some authorities have for longer courses of Phase 2 eradication therapy. The approach taken by the Pahang Melioidosis Registry [17] will in future permit an evaluation of adherence to the 12-20 week phase two treatment and its long term impact on late relapse. 

A specific issue raised by the emphasis on biosecurity is a need for post-exposure prophylaxis [27]. Though this has not been required for treatment of first responders to a bioterrorism incident involving *B. pseudomallei*, there are occasional accidental exposures in the clinical laboratory [28], and during field investigations of outbreaks when high bacterial concentrations may be encountered [29]. Consensus guidelines have been developed and rely on a combination of prompt risk assessment after the event, susceptibility testing of bacterial isolates, post-exposure prophylaxis with one or more of the preferred agents, and careful clinical monitoring [30]. Laboratory experiments with an animal model indicate that there is a 48hr window for administration of post-exposure prophylaxis [31]. However, this has probably done little more than slow disease progression in laboratory animals. There is therefore no reliable evidence to support the use or choice of Phase 0 prophylaxis regimens. 

The search for improvements in melioidosis treatment continues with the release of new antimicrobial agents. The new carbapenem Doripenem and the cephalosporin Ceftobiprole have both been shown to have bactericidal activity against *B. pseudomallei* in laboratory studies [32,33], but have yet to evaluated in clinical trials. Other promising new agents that may have a role in the treatment of melioidosis in future include Ceftalorine and Iclaprim, but assessment of their efficacy is at an earlier stage.

## 6. Conclusions

Melioidosis is a difficult infection to manage, not least because of its capacity to cause a rapidly fatal outcome despite the use of appropriate antibiotics. There has been modest progress in the last two decades, though mainly in the details of treatment delivery. At present, a universally effective and inexpensive treatment remains outside our grasp.
